# New Molecular Insights into the Inhibition of Dipeptidyl Peptidase-4 by Natural Cyclic Peptide Oxytocin

**DOI:** 10.3390/molecules24213887

**Published:** 2019-10-28

**Authors:** Veera C. S. R. Chittepu, Poonam Kalhotra, Tzayhri Osorio-Gallardo, Cristian Jiménez-Martínez, Raúl René Robles-de la Torre, Tzayhri Gallardo-Velazquez, Guillermo Osorio-Revilla

**Affiliations:** 1 Departamento de Ingeniería Bioquímica, Escuela Nacional de Ciencias Biológicas, Instituto Politecnico Nacional, Av. Wilfrido Massieu S/N, Col. Unidad Profesional Adolfo López Mateos, Zacatenco, Ciudad de Mexico 07738, Mexico; veerareddy9@gmail.com (V.C.S.R.C.); crisjm_99@yahoo.com (C.J.-M.); 2 Departamento de Biofísica, Escuela Nacional de Ciencias Biológicas, Instituto Politécnico Nacional, Prolongación de Carpio y Plan de Ayala S/N, Col. Santo Tomás, CP. Ciudad de Mexico 11340, Mexico; kalhotrapoonam@gmail.com (P.K.); gtzayhri@yahoo.com (T.G.-V.); 3 Departamento de Microbiologia e Immunologia, Facultad de Medicina Veterinaria Y Zootecnia, Universidad Nacional Autonoma de Mexico, Av. Universidad #3000, Delegacion Coyoacan, Col. Ciudad Universitaria, Ciudad de Mexico 04510, Mexico; tzayhriosoriogallardo@gmail.com; 4 Centro de Investigación en Biotecnología Aplicada CIBA, Instituto Politécnico Nacional, Carretera Estatal, Tecuexcomac-Tepetitla, Km 1.5, CP. Tlaxcala 90700, Mexico; rrenerdlt@yahoo.com

**Keywords:** diabetes treatment, cyclic peptides, oxytocin, dipeptidyl peptidase-4 inhibition, peptide therapeutics

## Abstract

Protease inhibition has led to treating many diseases and has been successful in producing many commercial drugs by pharmaceutical companies. Among many proteases, serine protease has been attractive in treating metabolic disorder diabetes mellitus (DM). Gliptins have been proven to inhibit dipeptidyl peptidase-4 (DPP4), a serine protease, and are an emerging therapeutic drug target to reduce blood glucose levels, but until now there is no natural cyclic peptide proven to inhibit serine protease DPP4. This study demonstrates the potential mechanism of natural cyclic peptide oxytocin (OXT) as a DPP4 inhibitor. To achieve this, initially, activity atlas and field-based models of DPP4 inhibitors were utilized to predict the possible features of positive and negative electrostatic, hydrophobic, and activity shapes of DPP4 inhibition. Oxytocin binding mode, flexibility, and interacting residues were studied using molecular docking simulations studies. 3D-RISM calculations studies revealed that the stability of water molecules at the binding site are favorable. Finally, an experimental study using fluorescence assay revealed OXT inhibits DPP4 in a concentration-dependent manner in a significant way (*p* < 0.05) and possess IC_50_ of 110.7 nM. These new findings significantly expand the pharmaceutical application of cyclic peptides, and in specific OXT, and implicate further optimization of OXT inhibition capacity to understand the effect of DPP4 inhibition. This work highlights the development of natural cyclic peptides as future therapeutic peptides to reduce glucose levels and treat diabetes mellitus.

## 1. Introduction

Proteases are associated with many signaling biological pathways, and it is has been proven that the number of diseases like cancer, HIV, infectious diseases, and diabetes are treated by inhibiting proteases [1,2]. Pharmaceutical companies develop commercial drugs to inhibit aspartyl proteases, serine proteases, and cysteine proteases. Among several protease inhibitors, serine protease inhibitors received significant interest in various applications focused mainly in therapeutics. Serine protease inhibitors shown in Table 1, are used in treating diseases like immune-related disorders [3], inflammatory [4], respiratory [5], AIDS [6], neurodegenerative [7], and metabolic disorders [8].

Features like therapeutic potential [9,10,11,12,13,14], good binding affinity, low toxicity, and quickly cleavable by proteolytic enzymes, make cyclic peptides potential future therapeutics [15]. Most of the cyclic peptides approved by the FDA (shown in Table 2) are used in bacterial [16] and fungal infections [17,18], oncology drugs [19], gastrointestinal disorders [20], and anemia [21,22]. Recently, cyclic peptides were proven to inhibit HIV-1 protease (aspartyl proteases) [23] and elastase, shedding light onto the use of cyclic peptides as protease inhibitors [24].

Phage display technology [25], Incretin-based cyclic peptide [26], cyclic peptides from mRNA display [27], and split-pool synthesis [28] are some of the tools used to develop cyclic peptide compounds. Recently computer-aided drug design technologies like molecular docking simulations are applied to identify, design rationally (called as de novo developed), and develop cyclic peptide ligand as leads to therapeutic drug targets of interest [29]. Till today, cyclic peptides as therapeutics in regulating glucose metabolism by inhibiting proteases have not been reported for the treatment of diabetes mellitus.

In Mexico, approximately 11.5 million people live with diabetes mellitus (DM), which is the main leading cause of acquired blindness, non-traumatic lower-limb amputations, and chronic kidney failure diseases [30]. Human dipeptidyl peptidase-4 (DPP4) is a serine protease, well known FDA-approved therapeutic drug target for regulating blood glucose metabolism and treating diabetes mellitus. Sitagliptin, Vildagliptin, Anagliptin, Saxagliptin, and Alogliptin are the main clinically used chemicals for human dipeptidyl peptidase-4 (DPP4) inhibition despite their side effects as pancreatitis, nausea, and anemia [31]. It is well known that incretin hormones glucagon-like peptide 1 (GLP-1) and glucose-dependent insulinotropic polypeptide (GIP) are responsible for insulin release and glucose regulation [32]. These hormones are inactivated by a serine protease human dipeptidyl peptidase-4 (DPP4). Since natural cyclic peptides are proven to inhibit various proteases, and to consider the beneficial aspects of cyclic peptides in comparison to small chemicals, like less toxicity, no accumulation in organs, and rapid degradation of peptides, we hypothesized that cyclic peptides from natural sources could be an alternative source of therapeutics to inhibit serine protease DPP4 for treatment of diabetes mellitus.

Among various naturally occurring cyclic peptides, oxytocin (natural cyclic peptide) [33] was chosen in this study to prove our hypothesis that, cyclic peptides possess serine protease inhibition activity, and specifically inhibit DPP4. To demonstrate our hypothesis, activity atlas and field-based models of DPP4 inhibitors from natural origin were utilized to elucidate molecular insights of oxytocin peptide into DPP4 inhibition. In addition, molecular docking simulations were used to understand the binding poses and interacting residues responsible for inhibitory activity. Also, the stability of water molecules at the binding site of oxytocin was studied using 3D reference interaction site model (3D-RISM) to gain deep insights into the binding of the cyclic peptide at DPP4 druggable region. Finally, fluorescence assay was used to demonstrate the potential role of oxytocin as a DPP4 inhibitor experimentally.

## 2. Results and Discussion

### 2.1. Computational Studies

#### 2.1.1. Activity Atlas and Field-Based Model Studies

**Figure 1:**
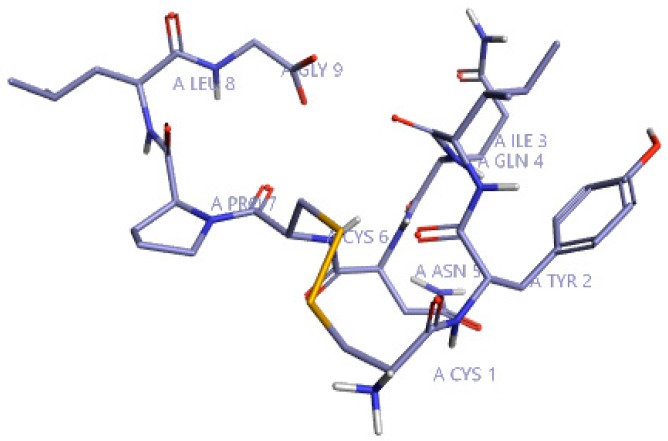
Three-dimensional structure of oxytocin visualized using Forge visualization software.

Initially, to understand the critical features of DPP4 inhibition of natural cyclic peptide oxytocin, the structure-activity relation (SAR) model constructed from previous studies by Kalhotra et al. (2018) was utilized [34]. Figure 1 shows the three-dimensional structure of oxytocin, whose sequence is CYS-TYR-ILE-GLN-ASN-CYS-PRO-LEU-GLY. Natural cyclic peptide oxytocin initially was aligned to the pharmacophore template representing natural DPP4 inhibitors, and then activity atlas model technology was utilized to understand how well the oxytocin fits the activity atlas model and field points regulating the DPP4 inhibition activity.

**Figure 2:**
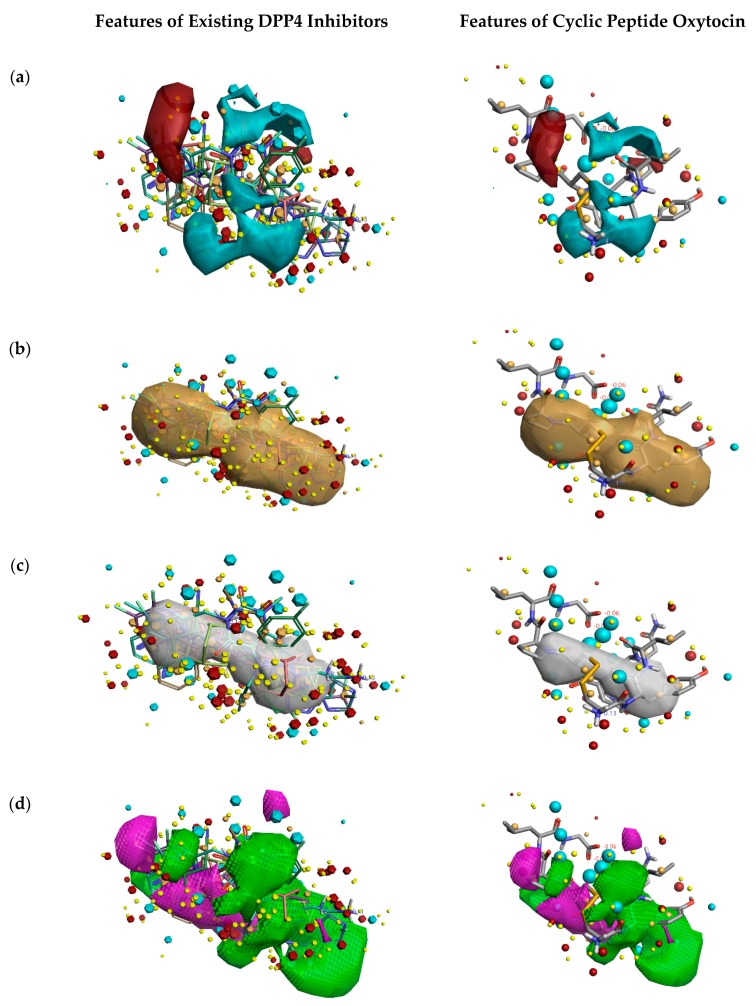
Activity atlas model describing the features responsible for DPP4 inhibition (left side) and features predicted for natural cyclic peptide oxytocin (right side). Forge tool was utilized to apply, analyze, and visualize features predicted by the activity atlas model. The generated model provides average electrostatics of positive actives in red color and negative electrostatics in blue color (**a**); an average of hydrophobicity (**b**); an average of actives (**c**); and activity cliff summary of favorable and unfavorable shapes (**d**).

Figure 2 describes the overall DPP4 inhibition features of exiting DPP4 inhibitors and features of cyclic peptide oxytocin, predicted by activity atlas model. The features like average activity cliff summary of electrostatics (shown in Figure 2a), the average activity of hydrophobics (shown in Figure 2b), and average activity of shapes (shown in Figure 2c) of oxytocin, which is responsible for DPP4 inhibition were visualized using the Forge visualization tool. Also, favorable and unfavorable regions responsible for DPP4 inhibition were examined using the activity atlas model and are shown in Figure 2d.

When field differences are examined between activity cliff summary of electrostatics (Figure 2a) of already proven DPP4 inhibitors and oxytocin peptide, the negative electron density spanning over proline, tyrosine, and cysteine amino acids of oxytocin are different from the constructed SAR model of DPP4 inhibitors. The decrease in the shape of actives and favorable regions of oxytocin in comparison to already proven DPP4 inhibitors (Figure 2c,d) might influence the inhibitory activity in a positive or negative manner. This could be addressed by experimental validation studies only.

#### 2.1.2. Molecular Docking Simulations

**Figure 3:**
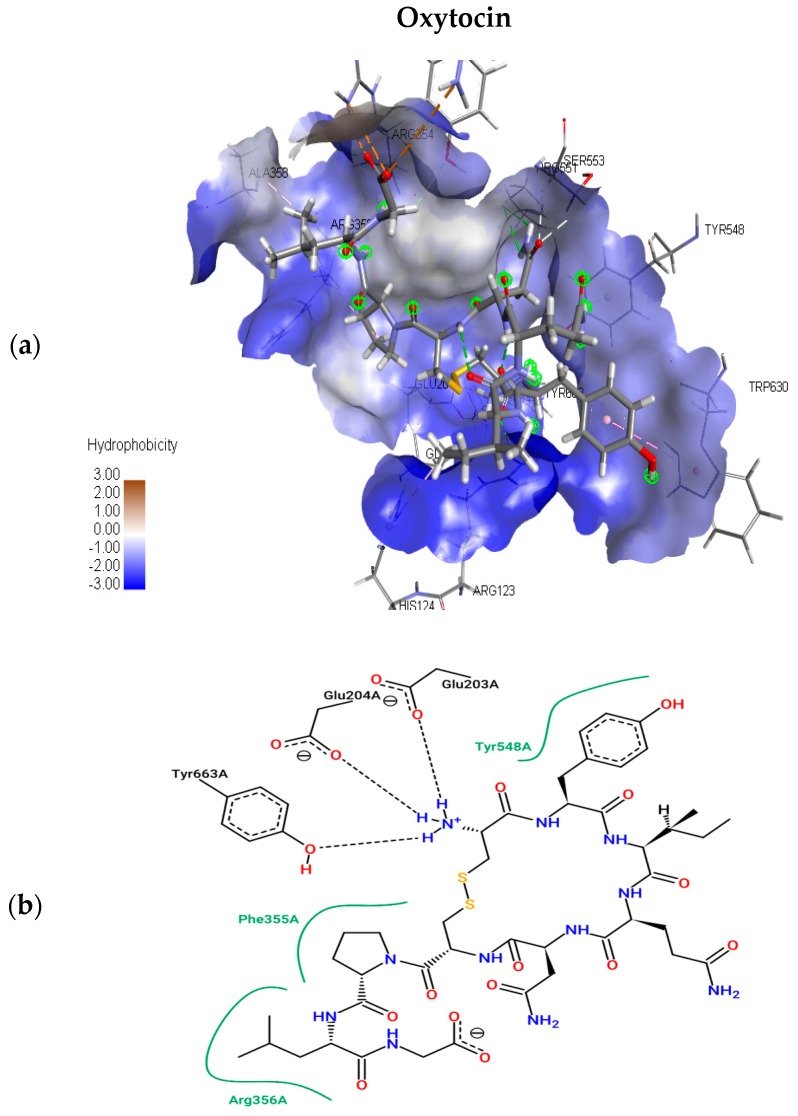
Top scoring binding pose views of oxytocin at the active site of DPP4 (**a**); and two-dimensional study of interacting residues (**b**), involved at the binding pose of oxytocin.

The present study utilizes molecular docking of cyclic peptides to understand the interactions between natural cyclic peptide oxytocin and the DPP4 receptor. The lead finder docking algorithm was used to understand the interactions. The docking simulations generated ten structure models of oxytocin bound DPP4 protein complexes. As a result, only the top-ranking and scoring model was further considered for studying binding pose view and interacting residues. Figure 3a represents the best scoring binding pose of oxytocin in complex with DPP4 with the highest score of −13.063 kcal mol^−1^. Figure 3b shows the interaction residues involved at the binding site of the best pose.

To understand the interacting residues at DPP4 active site upon binding by OXT, Pose View was utilized, and are ARG 356, PHE 355, TYR 663, GLU 204, GLU 203, and TYR 548. OXT binds to DPP4, and the interacting residues belong to hydrophobic S1 and S2 pocket, which have been proven to be responsible for the inhibition of DPP4. Further non-bonding interaction analysis of the DPP4 active site and oxytocin revealed the following: Pi-Alkyl interactions with residues HIS 124, and PHE 355; electrostatic interactions with GLU 203, GLU 204, and ASP 664. Upon the completion of binding studies, further research on understanding the stability of water molecules at the binding site of oxytocin-DPP4 was performed to gain insights into pockets and to predict the favorable and unfavorable region with water.

#### 2.1.3. 3D-RISM Analysis of Oxytocin-DPP4 Complex

**Figure 4:**
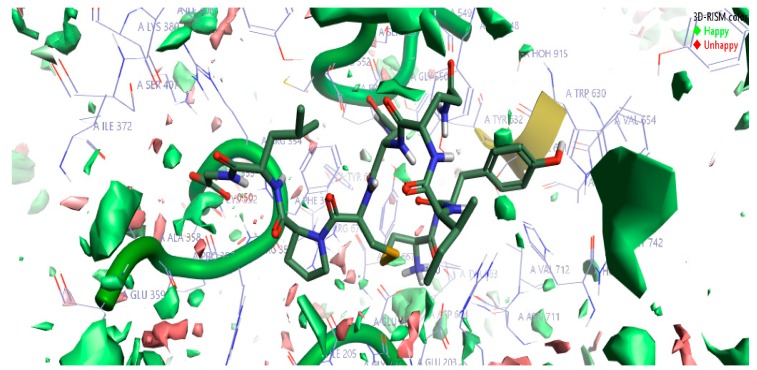
The stability of water molecules at the oxytocin bound to DPP4 protein calculated using the three-dimensional reference interaction model (3D-RISM). Free energy (ΔG) is colored in green for favorable water molecules and in red color for the unfavorable water molecules, calculated for the oxygen isodensity surface ρ = 5. Cresset visualization software was used to visualize the molecular insights of water molecules’ stability at the complex.

The top-scoring complex resulted from Flare docking algorithm studies was utilized to understand the stability of water molecules. The three-dimensional reference interaction model (3D-RISM) studies were carried out on oxytocin bound DPP4 protein. The high-water density of molecules was formed at the end of 3D-RISM calculations. Free energy ΔG was calculated for each water molecule, colored, and averaged in orientations shown in Figure 4. The density of oxygen surface of oxytocin bound to the DPP4 proteins was colored in green (favorable) and red (unfavorable). Favorable and unfavorable densities information led us to understand the stability of each water molecules at the active site. Figure 4 can help in gaining information to perform oxytocin modifications to improve the potency of DPP4 inhibition further.

### 2.2. Experimental Studies

**Figure 5:**
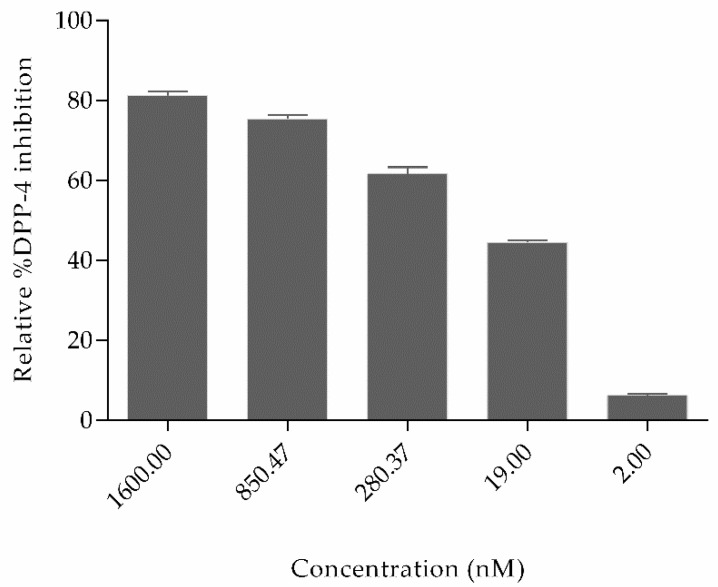
Relative percent inhibition of the dipeptidyl peptidase-4 (DPP4) enzyme by natural cyclic peptide oxytocin at five different concentrations. The two-tailed unpaired t-test was utilized to calculate the significant difference among different concentrations. Data represent mean ± S.D.

Figure 5 shows that the relative percent inhibition of DPP4 by OXT was 81.24%, 75.38%, 61.6%, 44.50%, and 6.3% for concentration 1600 nM, 850.47 nM, 280.37 nM, 19 nM, and 2 nM, respectively. The inhibitory concentration IC_50_ of oxytocin is 110.7 nM (shown in Figure S1). Based on the literature studies, some other natural non-cyclic peptides derived from amaranth, oat, buckwheat, high land barley, and milk proteins have already been proved to inhibit DPP4, which possess variable sequence length of 2–7 amino acids but do not contain cysteine–cysteine bonds. In comparison to oxytocin, the half maximal inhibitory concentration (IC_50_) of peptides derived from highland barley is 3.91 mg/mL; buckwheat is 1.98 mg/mL; oat is 0.99 mg/mL; whey protein is 1.08 mg/mL, and amaranth flour is 1.1 mg/mL [35,36,37]. Till today, Diprotin A peptide isolated from culture of Bacillus cereus possesses a IC_50_ value of 2.9 mg/mL [38] and peptides derived from various food hydrolysates possess IC_50_ values ranging from 3.5 to 2240 µM. (Peptide sequences and their IC_50_ values are shown in Supplementary Table S1) [39]. Even though several non-cyclic peptides from natural sources have been proved to inhibit DPP4, these results are the first evidence reported for the cyclic peptide oxytocin inhibiting dipeptidyl peptidase-4 and the inhibition is significantly effective.

Based on the obtained results, it clear that cyclic peptides have the potential to inhibit DPP4 enzyme in a concentration-dependent manner and that natural cyclic peptide oxytocin could be a promising lead for the development of DPP4 inhibitors.

**Figure 6:**
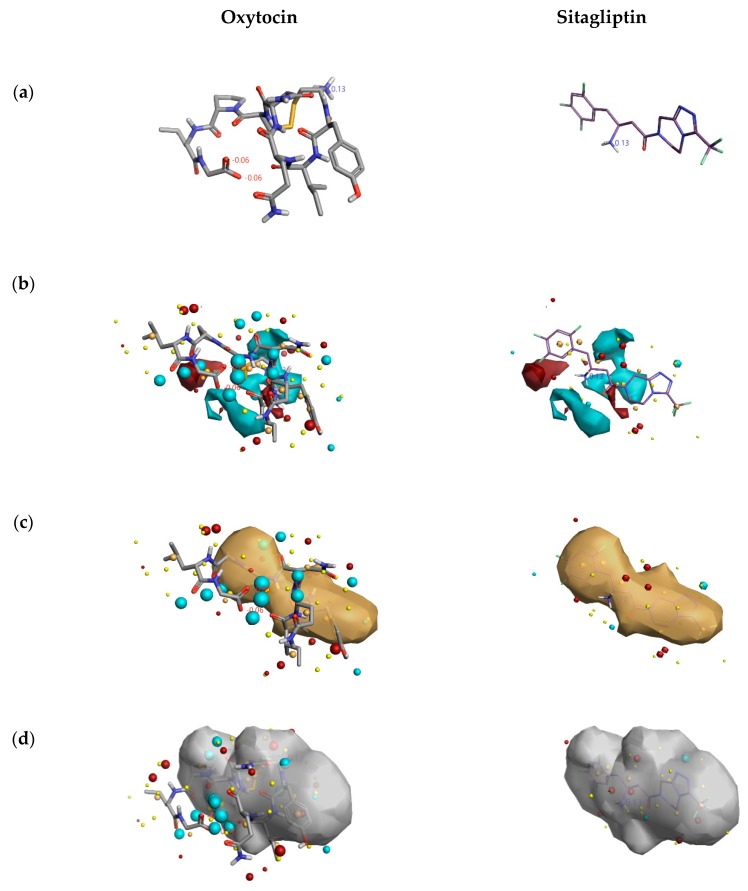
Comparison of features predicted by the activity atlas model on clinically used inhibitor and natural cyclic peptide oxytocin. The three-dimensional structure of oxytocin and sitagliptin (**a**); average activity summary of positive is shown in red color and negative electrostatics is shown in blue color (**b**); average shape of hydrophobics active (**c**); and average shape of actives (**d**) calculated using activity atlas model of DPP4 inhibitors. Forge visualization tool is utilized to understand the features.

From Figure 5, the DPP4 inhibitory activity (IC_50_) of oxytocin is less in comparison to sitagliptin. So, to gain insights into the lesser inhibition by oxytocin compared to sitagliptin, the activity atlas model features were utilized to understand the possible reasons. Figure 6 shows the comparison of features: positive electrostatics (red color), negative electrostatics (blue color), hydrophobic shapes (gold color), and activity of shapes (white color) of oxytocin with sitagliptin. Visual examination of all the fields revealed a significant increase in the electronegative feature of oxytocin in comparison to sitagliptin. Considering DPP4 inhibition assay and features predicted by the activity atlas model, it can be hypothesized that an increase in the electronegative field of oxytocin could be the potential reason for the less inhibitory activity of DPP4.

Even though the inhibitory activity of DPP4 by oxytocin is less than sitagliptin, it has been reported that oxytocin, a natural peptide, possesses multi-level effects like the regulation of glucose metabolism, enhanced insulin secretion, agonist OT-receptor signaling pathway, and weight control [40,41,42,43]. In addition, the levels of oxytocin have found to be reduced in the case of diabetes mellitus patients [44]. The results of the current work have shown that oxytocin also binds to DPP4 to inhibit protease activity, which could explain a possible mechanism of action to reduce blood glucose levels and increases the amount of insulin and glucagon-like peptide 1 levels in diabetes and obesity [45,46]. This result offers the unique therapeutic opportunity of oxytocin, and cyclic peptides in general, to have an essential role in the pharmaceutical landscape.

## 3. Materials and Methods

### 3.1. Materials

Oxytocin peptide was purchased from the company Parfarm S.A (District Federal, Mexico), and human dipeptidyl peptidase-4 inhibitor screening kit, Black-well 96 well plate for fluorescence assays were purchased from Sigma Aldrich (St. Louis, MO, USA).

### 3.2. Computational Methodology

#### 3.2.1. Field-Based and Activity Atlas Model Study

The field-based and activity atlas model constructed earlier using Forge software (Cresset Inc., Cambridgeshire, UK) was utilized in identifying leads for DPP4 inhibition [47]. In this study, oxytocin peptide was loaded into the constructed activity atlas model, and the Forge visualization tool was used to visualize the DPP4 inhibition features of oxytocin predicted by the constructed activity atlas model.

#### 3.2.2. OXT-DPP4 Molecular Docking Simulations

The three-dimensional structure of natural cyclic peptide oxytocin and DPP4 was retrieved from PDB ID 1NPO and PDB ID 4FFW, respectively. The Flare tool provided by Cresset Software (Cresset Inc., Cambridgeshire, UK) was utilized for protein preparation and to perform minimization. Chain A was chosen to perform molecular docking simulations using the Lead Finder algorithm provided by Flare software package of Cresset-group, to understand the cyclic peptide OXT binding site and interacting residues at DPP4 protein [48,49]. The binding site was chosen where the ligand sitagliptin is bound to DPP4, and then energy grid maps were calculated for the DPP4 binding site. The created energy map was then used to dock the oxytocin peptide. The Lead Finder’s scoring function was used to score the docked poses of oxytocin. Ten poses were generated for oxytocin peptide. The Discovery Studio Visualizer was used to study binding site interacting residues.

#### 3.2.3. 3D-RISM Analysis

Solvation studies were performed using the three-dimensional reference interaction site model (3D-RISM), the modern approach utilized to study the location and stability of water molecules in a protein. 3D-RISM uses the molecular Ornstein–Zernike equation to solvate peptide bound protein complex structure and provide 3D maps of correlation functions [50]. In this study, the density of solvent particles was extracted by keeping solute fixed and running infinite-time molecular dynamic simulations on the solvent.

3D-RISM calculations were carried out using Cresset’s XED force files on the high-resolution OXT peptide-DPP4 protein complex, to investigate the stability of water molecules bound at the binding site of the OXT peptide. The results of the calculations are displayed as iso-surfaces. The iso-surfaces are colored by ΔG. Negative ΔG values are colored with shades of green, characterized as favorable waters; these are the water molecules which 3D-RISM predicts to be more stable in DPP4 protein than in bulk water, and hence, more difficult to displace with oxytocin peptide. Similarly, the ΔG positive values are colored with shades of red characterized as unfavorable waters; these are water molecules which 3D-RISM predicts to be less stable in DPP4 protein than in bulk water, and hence are easy to displace with oxytocin peptide.

### 3.3. Experimental Methodology

The experimental studies were carried out using the human DPP4 inhibitor screening kit to validate the computational results. Briefly, the pure protein DPP4 cleaves non-fluorescence substrate H-Gly-Pro-AMC and releases fluorescence product AMC whose fluorescence emissions, λex = 360 nm and λem = 460 nm on each well, were recorded in a kinetic mode for 30 min using microplate reader Thermo Scientific Varioskan Flash Multimode Reader by Thermo Scientific. The relative percent of inhibition was calculated using Equation (1), where df/dt denotes changes in fluorescence with time. The graphs were plotted for all experiments (*n* = 3) using Graph Pad software. All groups were compared with *p* < 0.05 as statistically significant. To obtain the *p*-value, an ordinary one-way ANOVA unpaired t-test was applied using Graph Pad software (San Diego, CA, USA). All the data in graphs are expressed as mean ± S.D:

(1) $$\%\;Relative\;inhibition=\frac{\left(\frac{df}{dt}\right)DPP4\;enzyme-\left(\frac{df}{dt}\right)\left(DPP4-OXT\;peptide\right)}{\left(\frac{df}{dt}\right)DPP4\;enzyme}\times 100$$

## 4. Conclusions

The present work is the first to demonstrate the application of in silico studies like activity atlas model of DPP4 inhibitors, molecular docking simulations, and solvation studies to help in predicting the binding of the natural cyclic peptide at the druggable region of DPP4. In vitro studies revealed that natural cyclic peptides, like oxytocin, interact with DPP4 and inhibit the biological activity of DPP4 in a concentration-dependent manner with IC_50_ of 110.7 nM. The DPP4 inhibition potential of oxytocin offers new molecular insights and perspectives of oxytocin and cyclic peptides in general as lead to serine protease inhibitors and can be used as future therapeutics to reduce blood glucose levels and to manage diabetes mellitus.

## Figures and Tables

**Table 1:** Protease inhibitors in clinical use.

Company Name	Protease (Class)	Drug Name	Class
Novartis	Thrombin (Serine), Renin (aspartic)	Desirudin, Aliskiren	Peptidyl, Nonpeptidyl
Merck	DPP4 (Serine)	Sitagliptin	Nonpeptidyl
Bayer	Factor Xa	Rivaroxaban	Nonpeptidyl
Boehringer Ingelheim	Thrombin (Serine), HIV protease (aspartic)	Dabigatran, Tipranavir	Nonpeptidyl
GSK	Thrombin (Serine)	Argatroban	Nonpeptidyl
Bristol-Myers Squibb	ACE (Metallo)	Captopril	Peptidyl
Velcade	Proteosome (threonine)	Bortezomib	Peptidyl

**Table 2:** FDA approved cyclic peptides and their clinical use.

No.	Clinical Use	Name of Peptides
1	Bacterial and fungal infections	Telavancin, Dalbavancin, Oritavancin, and Anidulafungin
2	Oncology	Lanreotide, Romidepsin, and Pasireotide
3	Gastrointestinal disorders	Linaclotide
4	Anemia, chronic kidney disease	Peginesatide

## Supplementary Materials

The following are available online. Figure S1: The dose-response curve of oxytocin inhibiting DPP4 at different concentrations. Table S1: Peptides as DPP4 inhibitors.
