# Supplementary material for: New Molecular Insights into the Inhibition of Dipeptidyl Peptidase-4 by Natural Cyclic Peptide Oxytocin
Source: Molecules. 2019 Oct 28;24(21):3887. doi: 10.3390/molecules24213887 (PMC6864445; doi:10.3390/molecules24213887)
Supplement: Supplementary file 1 [file molecules-24-03887-s001.pdf]

Supplementary Data

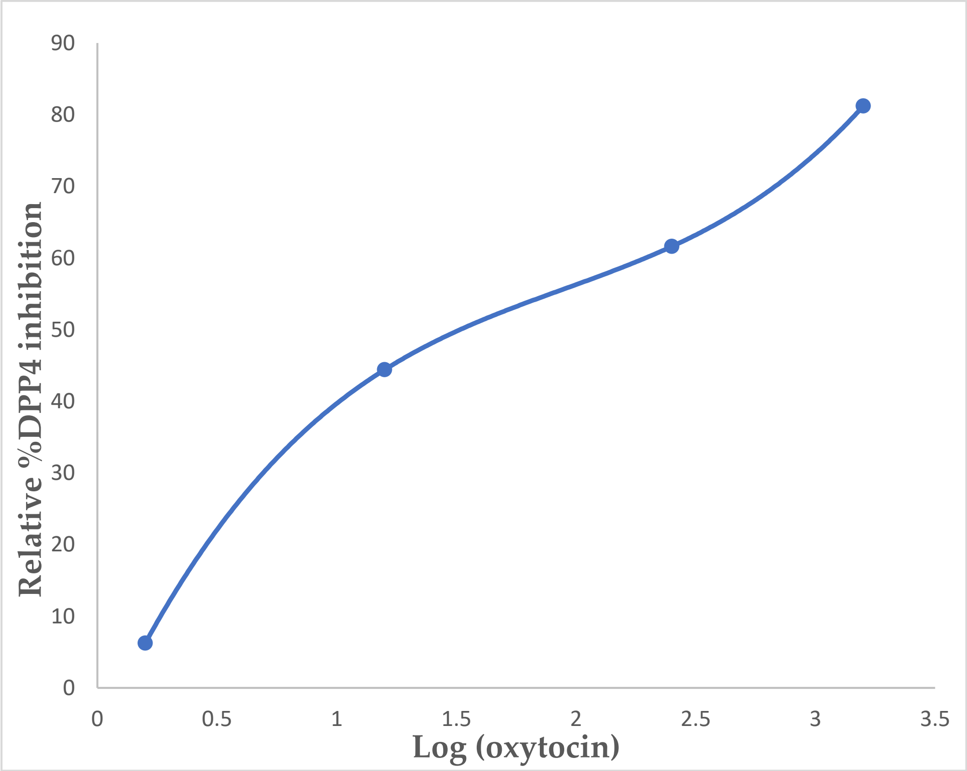

Figure S1. The dose-response curve of oxytocin inhibiting DPP4 at different concentrations.

Table S1. Peptides as DPP4 inhibitors.

Sl.No.	Peptide Sequence	IC50 (uM)
**1**	Diprotein A (IPI)	2.9
**2**	IPIQY	26.7
**3**	IPIY	35.2
**4**	WR	37.8
**5**	WK	40.6
**6**	GPAG	41.1
**7**	GPGA	41.9
**8**	WL	43.6
**9**	WP	44.5
**10**	IPAVF	44.7
**11**	LPQNIPPL	46
**12**	IPA	49
**13**	CAYQWQRPVDRIR	78
**14**	LPQ	82
**15**	LPYPY	90.8
**16**	PACGGFYISGRPG	96.4
**17**	PGVGGPLGPI GPCYE	116
**18**	HL	143
**19**	LPQNIPP	160
**20**	VA	168
**21**	LPL	186.8
**22**	YPYY	207.9
**23**	FPGPIPD	260
**24**	YPY	282
**25**	LPLPL	358
**26**	FL	399
**27**	IP	410
**28**	MP	870
**29**	VP	880
**30**	PGPIHDS	1000
**31**	IPPLQTPV	1300
**32**	RP	2240
